# Individual contextual factors in the validation of the Bernese pain scale for neonates: protocol for a prospective observational study

**DOI:** 10.1186/s12887-017-0914-9

**Published:** 2017-07-19

**Authors:** Eva Cignacco, Karin Schenk, Bonnie Stevens, Liliane Stoffel, Dirk Bassler, Sven Schulzke, Mathias Nelle

**Affiliations:** 1 Health Department, Midwifery Discipline, Bern University of Applied Sciences, Murtenstrasse 10, 3008 Bern, Switzerland; 2 Lawrence S. Bloomberg Faculty of Nursing and Faculties of Medicine and Dentistry, University of Toronto, Toronto, Canada; 3 Neonatalogy, Children’s Hospital, University Hospital of Bern, Bern, Switzerland; 4 Department of Neonatology, University Hospital Zurich and University of Zurich, Zurich, Switzerland; 5 Department of Neonatology, University of Basel Children’s Hospital (UKBB), Basel, Switzerland; 6 Department of Neonatology, Children’s University Hospital, Bern, Switzerland

**Keywords:** Pain assessment, Premature infants, Contextual factors, Diagnostic

## Abstract

**Background:**

The Bernese Pain Scale for Neonates (BPSN) is a multidimensional pain assessment tool that is already widely used in clinical settings in the German speaking areas of Europe. Recent findings indicate that pain responses in preterm neonates are influenced by individual contextual factors, such as gestational age (GA), gender and the number of painful procedures experienced. Currently, the BPSN does not consider individual contextual factors. Therefore, the aim of this study is the validation of the BPSN using a large sample of neonates with different GAs. Furthermore, the influence of individual contextual factors on the variability in pain reactions across GA groups will be explored. The results will be used for a modification of the BPSN to account for individual contextual factors in future clinical pain assessment in neonates.

**Methods and design:**

This prospective multisite validation study with a repeated measures design will take place in three university hospital neonatal intensive care units (NICUs) in Switzerland (Bern, Basel and Zurich). To examine the impact of GA on pain responses and their variability, the infants will be stratified into six GA groups ranging from 24 0/7 to 42 0/7. Among preterm infants, 2–5 routine capillary heel sticks within the first 14 days of life, and among full-term infants, two heel sticks during the first days of life will be documented. For each heel stick, measurements will be video recorded for each of three phases: baseline, heel stick, and recovery. The infants’ pain responses will be rated according to the BPSN by five nurses who are blinded as to the number of each heel stick and as to the measurement phases. Individual contextual factors of interest will be extracted from patient charts.

**Discussion:**

Understanding and considering the influence of individual contextual factors on pain responses in a revised version of the BPSN will help the clinical staff to more appropriately assess pain in neonates, particularly preterm neonates hospitalized in NICUs. Pain assessment is a first step toward appropriate and efficient pain management, which itself is an important factor in later motor and cognitive development in this vulnerable patient population.

**Trial registration:**

The study is registered in the database of Clinical Trial gov. Study ID-number: NCT 02749461. Registration date: 12 April 2016.

## Background

In order to ensure their survival, premature born infants hospitalized in a neonatal intensive care unit (NICU) are subjected to many painful diagnostic and therapeutic procedures [1–3]. Although there have been efforts in recent years to quantify, and most importantly, reduce the number of procedural exposures to pain in preterm infants, procedural acute pain remains a challenge in the NICU setting [3–5]. Often, these treatment interventions take place during a crucial period in the development of the nociceptive and central nervous systems [6–8]. There is more and more alarming evidence that repeated painful stimuli at this early age may induce both structural and functional reorganization of the nervous system [7, 9–13] and result in an altered pain response [14–16]. As a consequence, the motor and cognitive development of premature infants may be impaired [9, 13, 17–22]. In premature infants requiring intensive care, the frequency of exposure to pain and systematic implementation of preventive pain measures are therefore of key importance for their later development [4, 5]. Accurate pain measurement is the first step toward effective pain management.

### Pain assessment in neonates

Clinical pain assessment in neonates, particularly those delivered preterm, is highly challenging [4, 23]. In the clinical setting, their pain responses have to be observed and assessed using behavioral and physiological indicators, which can vary across premature infants depending on their physiological and neurological development stages [23]. Behavioral indicators used as pain assessment tools include body movements, facial expressions and crying [24]. Some pain assessments also include behavior status indicators, e.g., sleep-wake state [25, 26]. Physiological responses to pain include, for instance, changes in heart rate, respiratory rate, blood pressure, oxygen saturation, vagal tone, and peripheral blood flow [25, 27]. Recently, researchers have begun to investigate more objective approaches to pain assessment, such as measurement of heart rate variability, skin conductance and cortisol as a biomarker of stress [23, 25]. To better understand and assess neonatal pain responses at cortical level, newer brain-oriented techniques, such as electroencephalography (EEG) [28, 29] and functional magnetic resonance imaging (fMRI) [30, 31], are used [11, 32–34]. However, for systematic clinical pain assessment, exclusively observable indicators need to be considered.

Because of the complex nature of pain, multidimensional pain measures that include behavioral and physiological indicators are generally assumed to be most appropriate for the clinical setting [23]. Although most infants show both types of pain response indicators, the correlation between these two indicators is often low [25, 35]. Moreover, no consistent associations between behavioral, physiological and cortical measures of pain have been detected so far [36]. In the face of inconclusive associations between different indicators of pain, the validity of existing multidimensional tools and their choices of indicators are currently being questioned, and, to date, no universally accepted gold standard exists for neonatal pain assessment [23].

More than 40 pain assessment scales for premature and full-term infants exist to date [25, 37]. The majority were designed for research purposes and are inappropriate for routine clinical procedures (e.g., because they require extended observation periods) [25, 38]. Furthermore, only a few have undergone extensive psychometric testing and are both reliable and valid [25, 39]. Of the pain assessment scales compiled for clinical application, few have been validated in premature infants and even fewer consider individual contextual factors, e.g. gestational age (GA) and health status [23, 40].

### The Bernese pain scale for neonates

The Bernese Pain Scale for Neonates (BPSN; [41]) was developed by nurses of the University Hospital of Berne primarily for clinical use. Since its development in 1996, it has been widely used for bedside pain assessment in NICUs in the German speaking areas of Europe. Several hospitals in Switzerland have fully integrated the BPSN into their daily routine.

The BPSN is a 9-item multidimensional pain assessment tool that includes behavioral and physiological indicators. The instrument consists of seven subjective (alertness, crying, consolation, skin color, facial expression, posture, and changes in respiratory rate) and two physiological (i.e. objective) (changes in heart rate and oxygen saturation) indicators. Each item is rated on a four point Likert scale (0, 1, 2, and 3). Higher scores indicate greater pain-related distress, and a total score of 11 or higher is considered to indicate pain.

In the year 2004, the BPSN was validated to differentiate between pain and non-pain status in neonates between 27 and 41 weeks of gestation [41]. The results suggested that the BPSN is a valid and reliable pain assessment instrument for assessing acute pain in term and preterm neonates. A shortcoming of this first validation study of the BPSN is the small study population of 12 infants. Furthermore, increasing evidence indicates that pain reactions of neonates are probably influenced by more than noxious stimulation alone; individual contextual factors might also impact pain reactivity [40, 42–44]. Currently, the BPSN focuses entirely on physiological and behavioral indicators.

### Individual contextual factors

Individual contextual factors encompass individual infant characteristics (e.g., GA, gender, health status, and weight), previous pain experience, or the duration of hospitalization [23, 44]. The variability in pain responses between and within premature infants as well as the low association between behavioral and physiological pain responses may be explained by the influence of individual contextual factors [35, 42, 45, 46].

Neonatal age is the most commonly examined individual contextual factor associated with neonatal pain response [44]. Premature neonates generally seem more sensitive to painful stimulation than full-term newborns. In addition to having low reflex thresholds [47, 48], newborns lack the inhibitory control that mature brain structures would exert [49]. As a result, premature neonates display diffuse responses to noxious stimuli rather than more complex affective reactions [50]. Moreover, the association between behavioral and physiological stress responses may differ depending on GA [35]. Although older GA infants displayed a positive association between the extent of behavioral pain reaction and heart rate levels, Lucas-Thompson et al. (2008) found no association between physiological and behavioral responses in the youngest GA infants. Despite the high variability in behavioral and physiologic pain responses in premature neonates, their responses are less intense [42, 45, 51, 52].

The results of several studies suggest that facial expression in response to pain increases with GA [45, 52–55]. This difference is manly influenced by the older infants’ increased facial expressiveness, which results from their more developed nervous system and facial muscles [53, 54]. In contrast, several studies have reported no significant relationship between GA and facial expression in response to pain [44, 56]. However, the consideration of reduced facial movement in response to pain in premature neonates is important. Using pain assessment scales which rely only on facial expressions may lead clinicians to the incorrect conclusion that younger premature infants do not feel or feel less pain [57]. In addition, the presence of endotracheal tubes in premature neonates impedes using facial reaction and crying as indicators of pain because endotracheal tubes are typically secured by taping them to the skin of the face [52, 54, 57]. Therefore, the consideration of other behavioral pain indicators encoded in specific body movements (e.g., hand on face), may provide further information about pain in premature infants with extremely low GA [52, 56, 58].

Several studies have examined the influence of previous pain exposure on reaction to pain, but the findings do not provide a clear answer [44]. Some studies report that infants subjected to frequent painful procedures during their hospitalization display less intense behavioral responses to heel sticks than those who have undergone fewer procedures [46, 52, 59]. The dampened pain responses in very premature neonates may be a sign of exhaustion or a state of passivity resulting from the numerous procedures they experience during their stay in a NICU [43, 60, 61]. Contrary to those findings, other studies suggest that repeated exposure to pain may lead either to increased pain response (hyperalgesia) or to pain responses without painful stimulus (allodynia) [15, 62].

Few studies have investigated the influence of other contextual factors (e.g., gender, health status) on pain reactions in neonates, and of those that have, the results are inconsistent [44]. This might be explained by methodological limitations (e.g. the comparison of different GA groups and the use of a variety of pain assessment tools) [44]. One challenge in examining the influence of contextual factors on pain response is the associations between the individual factors [44]; for example, extremely low GA infants have a longer stay in a NICU and are exposed to a higher number of painful procedures than more mature infants. Due to the fact that contextual factors can lead to underestimation or misjudgment of pain severity [54, 63–65], further research is needed to better understand the factors that influence pain responses in neonates. Relevant contextual factors should also be considered in future pain assessment.

### Study aims

The aim of this observation study is the validation of the BPSN, using a large sample of neonates spanning a full range of GAs. The validation will involve the detection of the underlying structure of the data and the examination of the concurrent validity of the BPSN with the Premature Infant Pain Profile-Revised (PIPP-R; [26]), construct validity, interrater reliability, specificity and sensitivity. Furthermore, the variability of pain reactions over time related to behavioral and physiological patterns will be analyzed and the relationship between behavioral and physiological indicators examined. In addition, the influence of contextual factors on the variability of pain reactions across GA groups will be explored. Finally, the results of this analysis will be used for modification of the BPSN, to account for individual contextual factors in future clinical pain assessment in neonates.

Based on a previous validation study of the BPSN [41], we hypothesize that the BPSN will be a valid and reliable pain assessment tool for premature and term infants. In addition, we expect that the impact of single contextual factors on infants’ pain reaction will be described and considered for future pain assessment. In particular, we anticipate finding a difference in pain reaction depending on GA. Moreover, we hypothesize that behavioral and physiological indicators will show low association across time and that this low association may be explained by the influence of individual contextual factors.

## Methods

This prospective multisite validation study focuses on psychometric testing of the BPSN and involves repeated measurement design. The study will take place in three university hospital NICUs in Switzerland (Basel, Bern and Zurich).

**Fig. 1:**
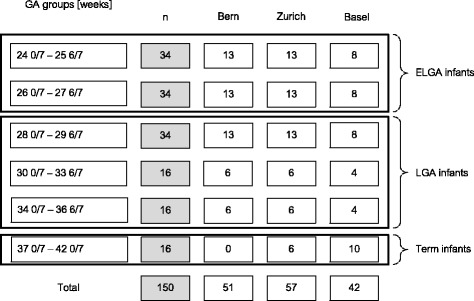
Stratification of sample according to gestational age (GA) and expected sample numbers (n) (ELGA = extremely low gestational age; LGA = low gestational age)

In total, 150 preterm and healthy-term infants hospitalized in a NICU will be included. Consecutive sampling will be used to recruit subjects and the infants will be stratified according to GA at birth (Fig. 1). Stratification is based on the assumption that premature neonates with a lower GA will show a higher variability in pain responses, due to their neurological immaturity, than will premature neonates with a higher GA and full-term infants [42]. Therefore, larger sample sizes of premature infants with GAs between 24 0/7 and 29 6/7 weeks (*n* = 102) will be included, compared to the samples of those with GAs between 30 0/7 and 42 0/7 weeks (*n* = 48).

### Inclusion and exclusion criteria

Premature infants born between 24 0/7 and 36 6/7 weeks of gestation will be included if they are expected to undergo 2–5 routine capillary heel sticks during the first 14 days of life. Full-term infants born between 37 0/7 and 42 0/7 weeks of gestation will be included if they are expected to have at least 2 routine capillary blood samplings during their first days of life. Furthermore, signed consent is needed from the infant’s parents, who have to understand either German or French.

Infants will be excluded if they have suffered a high-grade intraventricular hemorrhage (grades III and IV), if they have a severe life-threatening malformation or suffer from any condition involving partial or total loss of sensitivity, if they have had an arterial cord pH < 7.15, if they have had surgery for any reason, or if they have a congenital malformation affecting brain circulation and/or cardiovascular system. Infants treated with continuous positive airway pressure (CPAP) or mechanical ventilation will be included if they meet the other inclusion and exclusion criteria.

### Recruitment and data collection procedures

In each study center, a trained study assistant will identify potentially eligible infants and inform the parents about the study both verbally and via printed information material. Interested parents will receive the information material and a copy of the informed consent form to read. A member of the research team will answer any parental questions about the study. No study procedures will be performed until a signed informed consent form is obtained from the child’s parents.

After written consent has been received, the neonate will be videotaped (using a HC-V757 high-definition camcorder manufactured by Panasonic, Osaka, Japan) during his or her next 2–5 routine capillary heel sticks. Before each heel stick procedure, every infant will receive a dose of 24% oral sucrose (0.2 ml/kg bodyweight) as a pain relieving intervention in accordance with standards of care [66]. Video sequences and physiological variables will be recorded continuously from 2 to 3 min before the beginning of the heel stick procedure (baseline phase), through the heel stick (heel stick phase) and until 2–3 min after the heel stick (recovery phase). Therefore, three rating sequences will be produced for each heel stick. The camera operator will begin each video sequence by focusing on the face of the neonate for at least one minute to allow adequate assessment of facial activity and cry. Then, the infant’s body will be recorded for another minute. For healthy-term infants, six video sequences per infant will be produced, resulting in 96 videos (2 heel sticks * 3 phases * 16 n). For premature neonates, 2010 video sequences (5 heel sticks * 3 phases * 134 n) will be produced. This will lead to a total of 2106 video sequences, all of which will be filmed by trained study collaborators. Each video sequence will be checked for quality, and digitally elaborated by trained study assistants using Final Cut Pro X (Apple Inc., Cupertino, CA, USA) video editing software. To preserve rater blindness, any information that could indicate the heel stick phase to the raters will be eliminated. Data quality and completeness of the video sequences will be controlled continuously by the doctoral student before uploading each video sequence onto a web-based rating tool. The web-based rating tool has been developed specially for the study and includes a randomizing generator. Uploaded sequences are randomized related to sequence number, phases and presentation order. Five trained nurses who are presently working in a NICU and are experienced users of the BPSN will retrieve the randomized sequences from the web-based platform and will rate the behavioral pain reaction by means of the BPSN and the PIPP-R.

Individual contextual factors will be retrieved retrospectively from patient charts by trained study assistants. All extracted data will be entered into secuTrial®, a web-based data capture system (InterActive Systems, Berlin, Germany). Five percent of the patient charts will be audited by the doctoral student to detect and correct discrepancies. Emerging questions and inconsistencies during the overall data collection process will be continuously discussed to ensure the quality of ongoing data extraction.

### Measures

To establish concurrent validity, neonates’ pain expression is measured by the BPSN [41] and the PIPP-R [26]. The BPSN measures 9 indicators. The two physiological indicators will be captured on an ongoing basis from the neonate’s routine continuous monitoring records (heart rate and oxygen saturation) during the video recording. The six subjective indicators (sleeping state, crying, consolation, skin color, facial expression, posture, and breathing) will be rated by five independent and blinded video raters on a 4 point Likert scale. The raters are blinded towards the phase of the video sequence they are looking at (baseline, heel stick, and recovery). The PIPP-R, which is widely used in North America for assessing acute pain in neonates, measures five indicators of which two are physiological (heart rate and oxygen saturation). The three behavioral indicators (brow bulge, eye squeeze, and naso-labial furrow) will also be assessed by the five raters. Each indicator of the PIPP-R is numerically rated on a Likert scale from 0 to 3 points, with higher ratings reflecting the rater’s impression of more intense pain responses. Additionally, the PIPP-R accounts for GA and baseline behavioral states as contextual factors. According to the instructions of the authors, these contextual factors need only be scored if there are changes in any of the behavioral or physiological items [26]. Neonates with the youngest GAs and those in quiet sleep receive the highest scores for these indicators. The PIPP-R scores will be used as a standard reference in this study.

Based on the findings of a systematic review [44], the following individual contextual factors will be retrieved from patient charts: demographic contextual factors, including GA at birth, gender, birth weight, nationality, parity and way of delivery; the primary diagnosis and the most common comorbidities in preterm neonates, including bronchopulmonary dysplasia, necrotizing enterocolitis, respiratory distress syndrome, patent ductus arteriosus, septic events, cardiac events and respiratory events; the health status at time of birth measured by the Clinical Risk Index for Babies (CRIB; [67]). For the time of each heel stick, the following individual contextual factors will be retrieved: postnatal age; post-menstrual age (GA at birth combined with postnatal age); weight; CPAP or mechanical ventilation at the time of the heel stick procedure; medication administered (sedatives, opioids, non-opioids, steroids, caffeine, antibiotics and catecholamines) from birth and between the recorded heel stick procedures; number of previous painful (e.g., heel stick) and non-painful (e.g., diaper change) interventions from birth and between the recorded heel stick procedures (painful and non-painful interventions were defined in a previous study [68]); number of painful and non-painful procedures in the past 24 h; time since the last painful and non-painful interventions; and, finally, type of last painful and non-painful interventions. The duration of each heel stick and the number of additional sucrose doses given during the heel stick procedures will be registered while video recording.

### Data analyses

Data will be analyzed using SPSS (IBM© SPSS© Statistics Version 23.0, IBM Corp, Armonk, NY, USA) and Stata (Stata/MP 13.1, StataCorp LP, Lakeway Drive, USA). Initially, an exploratory analysis will be conducted to describe the data and uncover any anomalies that may impact the validity of the data analysis. Methods for handling missing data will be applied after considering the volume and pattern of missing data. Descriptive statistics including measures of central tendency and dispersion will be used to characterize the individual variables and to determine the distribution of the data.

Several data analyses will be used for the validation of the BPSN. An exploratory factor analysis will be performed to analyze the underlying structure of the data. Cronbach’s Alpha and item-total correlations will be conducted to analyze the reliability of the scale. Furthermore, construct validity will be examined by comparing mean measurements at each of the three rated phases (baseline, heel stick and recovery). The analysis will be performed for the total sum score of the BPSN as well as for the physiological items and the behavioral items alone. In order to determine the concurrent validity of the BPSN with the PIPP-R, the total sum scores of the two tools will be correlated. Intra-class correlation (ICC) will be used to determine interrater reliability across the 2–5 heel sticks. To test sensitivity and specificity in the BPSN, a receiver operating characteristic (ROC) curve analysis will be performed using the PIPP-R as reference value. Furthermore, the pain and non-pain cut-off values of the two instruments will be compared.

To explore and depict both temporal variability of pain reactivity between measurements of each subject, and variability between corresponding measurements of all subjects, linear mixed modeling will be applied to the behavioral and physiological data on pain reactivity. Additionally, individual contextual factors will be added to these models to test for associations with the BPSN scores. As contextual factors are highly dependent on organizational procedures, the possible confounding effect of the participating sites will also be taken into account.

In addition to analyzing the total sum scores of the BPSN, the separate physiological and behavioral subscores will be tested both against the total scores and against one another. Pearson correlation will be used as a descriptive indication of the strength of associations, while linear mixed modeling will be used to test the associations themselves.

### Sample size and power

The target sample size of 150 neonates is indicated on a power analysis of the hypothesized association between the BPSN and GAs at baseline. This analysis is based on the data from a descriptive-explorative analysis (*n* = 23) and a previous study (*n* = 71; [69]), i.e., assuming an alpha of 0.05, a beta of 0.80, with at least three baseline heel sticks conducted per study infant (taking into account both intra- and inter-infant variability). Because an attrition rate of 10–15% is anticipated, approximately 170 infants will be enrolled in the study.

## Discussion

The BPSN is already widely used in clinical settings in the German speaking areas of Europe. Pain assessment with the BPSN requires only two to three minutes of observation. Despite its practical application, another advantage of the BPSN is its consideration of various aspects of behavioral pain responses. Because of less intense facial reactions in premature neonates and the frequent presence of artificial respiration in this patient population, the consideration of various behavioral indicators of pain may provide further information for appropriate pain assessment. In addition, the repeated measurement design in this study will facilitate consideration of the development of pain responses across time.

The validation of the BPSN on a large sample of neonates with different gestational age and the consideration of the influence of individual contextual factors on pain reactivity should lead to a higher accuracy of routine pain assessment. A revised version of the BPSN may help the clinical staff to prevent and minimize the pain endured by neonates, particularly preterm neonates in NICUs. For preterm infants requiring intensive care, appropriate and efficient pain management is an important factor in later motor and cognitive development. This study will hopefully contribute to a more accurate pain assessment tool and to the prevention of negative long-term outcomes in this vulnerable patient population.

## Abbreviations

BPSN: Bernese Pain Scale for Neonates
GA: Gestational age
NICU: Neonatal intensive care unit
PIPP-R: Premature Infant Pain Profile-Revised
